# Differences in Personal, Familial, Social, and School Factors Between Underachieving and Non-underachieving Gifted Secondary Students

**DOI:** 10.3389/fpsyg.2019.02367

**Published:** 2019-10-22

**Authors:** Raquel Gilar-Corbi, Alejandro Veas, Pablo Miñano, Juan-Luis Castejón

**Affiliations:** Department of Developmental Psychology and Didactics, University of Alicante, San Vicente del Raspeig, Spain

**Keywords:** gifted students, underachievement, identification methods, academic achievement, individual characteristics, social characteristics, parent involvement

## Abstract

Using various identification methods, differences between underachieving and non-underachieving gifted students in personal, familial, social, and school variables were analyzed in a sample of 164 gifted students with IQs of 120 or higher; the sample was drawn from a larger sample of 1,400 compulsory secondary education students. Three procedures for identifying underachieving students were used: the standardized difference method, the regression method, and the Rasch method. The different profiles of underachieving and non-underachieving students in the personal, familial, social, and school variables were compared using MANOVA and ANOVA tests. Results revealed that underachieving gifted students scored significantly lower in learning strategies, goal orientations, self-concept, attitudes toward teachers, and perceived parent involvement in school variables. These results have clear educational implications as a result of identifying differences in non-cognitive factors.

## Introduction

In the field of education, the term underachievement has received increasing attention in recent decades. It provides methods for both its detection and the correct identification of the cognitive and non-cognitive variables involved (Lau and Chan, 2001; McCoach and Siegle, 2003a, b, 2011; Matthews and McBee, 2007). The first issue concerning underachievement is the definition, given the fact that there is no consensus on it (McCoach and Siegle, 2011). From the scientific literature, it is clear that underachievement refers to students whose achievement is lower than expected based on their cognitive abilities (McCoach and Siegle, 2003b; Phillipson, 2008).

Underachievement studies, especially in the United States (US), have traditionally focused on gifted students (Reis and McCoach, 2000; Obergriesser and Stoeger, 2015), whereas those in China have considered all ranges of ability (Phillipson, 2008, 2010; Dittrich, 2014).

The three statistical methods conventionally used for identifying underachieving students include the absolute split method, the simple standardized difference method, and the regression method (Lau and Chan, 2001; McCoach and Siegle, 2011). For the absolute split method, discretionary cut-off scores are used for the highest mental ability (for example, the highest 5%) and the lowest academic performance (for example, the lowest 5%) once the punctuations have been converted into standard scores. The simple standardized difference score method analyses the distance between the standardized performance score and the standardized ability score. If this distance exceeds the discretionary margin (usually 1 standard deviation or SD), a student can be considered as underachieving (*d* < −1) or overachieving (*d* > 1). McCall et al. (1992) pointed out that the simple standardized difference score method can produce overestimation of these types of students in the high and low ability ranges. One of the most common methods for identifying underachievement is the regression method (Lau and Chan, 2001; McCoach and Siegle, 2011), which analyses the deviations of students’ scores from the regression line of the measure of performance according to the measure of capacity. These statistical methods are based on the use of arbitrary cutoffs, as well as the use of standardized transformations that do not suppose the assumption that the original data are interval in nature (Fletcher et al., 2005; Phillipson, 2008) and generate a uniform percentage of underachieving students (Plewis, 1991; Ziegler et al., 2012).

To improve the objective use of the interval scale, the latest method employed in identifying underachieving students is the Rasch model (Phillipson and Tse, 2007; Phillipson, 2008). This model supposes that the probability of a given subject/item interaction is only controlled by the difficulty of the item and the ability of the subject, which are conditioned by the item situations of the supposed latent variables along the same scale structure (Wright and Stone, 1979; Rasch, 1980; Bond and Fox, 2007). Therefore, using the same measurement scale establishes homogeneous intervals, implying the same differences between item parameters and person ability and therefore the same probability of success (Preece, 2010). The adjustment of this interaction can be performed by employing residual measures and standardized punctuations for a specific item or subject (Bond and Fox, 2007).

Veas et al. (2016a) compared the statistical methods employed for detecting underachievement (the standardized difference method, the regression method, and the Rasch method) in a sample of 1,182 first- and second-year secondary students from eight secondary schools in Spain. The results showed varying percentages of underachieving students that included 14.55% (simple standardized difference), 15.39% (regression method), and 30.37% (Rasch model), depending on the statistical method employed; boys showed higher percentages (65%) than girls.

### Theoretical Framework

During the last years, important advances have been made to understand underachievement as an integrated and explanatory model, especially from the gifted education perspective. In this context, the actiotope model of giftedness (Ziegler, 2005) constitutes an appropriate framework that tries to explain how external and internal variables relate to each other.

Ziegler and Stoeger (2017) use the term “actiotope” to consider a student as the unit of analysis. An actiotope can be defined as a dynamic and personal perspective in a specific environment. Exogenous resources are important to build actiotopes’ action repertoires in educational contexts. Concretely, when exogenous resources enter the actiotope, they are referred to as educational capital (Ziegler and Baker, 2013). Educational capital is defined as all the resources that can be used to promote learning. Five types of educational capital have been proposed: economic educational capital (wealth, possessions, money, or valuables that can be invested), cultural educational capital (value systems, thinking patterns, and models), social educational capital (people and social institutions), infrastructural educational capital (materials implemented in learning), and didactic educational capital (design and improvement of education and learning processes).

Additionally, endogenous resources also affect individual functioning, which is called learning capital. Again, these resources are organized into five types: organismic learning capital (a person’s physiological and constitutional resources), telic learning capital (a person’s anticipated goal states that satisfy their needs), actional learning capital (the totality of actions that a person is able to perform), episodic learning capital (the simultaneous goal- and situation-relevant action patterns that are accessible to a person), and attention learning capital (the quantitative and qualitative attentional resources that a person can apply to learning).

### Personal Factors Involved in Underachievement

Regarding personal factors, Colangelo et al. (2004) found that using self-regulation strategies, learning strategies, and study techniques explicate the differences between high achievement and low achievement in high-ability students.

Studies from the US and China have detected minor levels of motivation associated with underachievement (Schick and Phillipson, 2009; Dunlosky and Rawson, 2012). McCoach and Siegle (2003a) found that gifted underachieving students differed in their school attitudes, attitudes toward teachers, motivation, self-regulation, and valuation objectives.

Meanwhile, the role of self-concept in the underachievement process is not clear (Preckel and Brunner, 2015). Several studies have reported poorer academic self-concept in underachievers (Rimm, 2003) and poorer general self-concept but not poorer academic self-concept in gifted underachievers (McCoach and Siegle, 2003a).

Castejón et al. (2016) explained the different learning strategies, goal orientations, and self-concepts of overachieving, normally achieving, and underachieving students in secondary education using a sample of 1,400 Spanish students. The results indicated that overachieving students reported significantly better scores than underachieving students in learning strategies and goals, academic self-concept, personal self-concept, relationship with parents, honesty, and personal stability. Along the same lines, Heyder et al. (2017) analyzed the variables involved in underachievement in boys’ language skills, finding that self-concept, motivation, previous performance, and family characteristics were key variables in the explanation of underachievement.

### Family and Social Factors Involved in Underachievement

Regarding family and social factors, the results obtained by Phillipson (2010) showed the relevance of these factors in the academic achievement of children, despite the children’s intellectual capacities. In high- and medium-ability students, parental expectations influenced the students’ achievement through the students’ ability, while in low-ability students, parental expectations influenced students’ achievement in a direct way. There are some studies analyzing parental influence on the achievement or underachievement of their children (Rimm and Lowe, 1988; Yazdani and Daryei, 2016). Certain patterns of familial settings may be related to underachievement (Baker et al., 1998; Rimm and Lowe, 1988). Parents of high-performing students show interest in academic achievement, while parents of underachieving students often show disinterest in school and education.

Reis and McCoach’s (2000) review of family factors showed that most studies of underachieving students focus on gifted students’ family structures and environments; parents’ involvement is highly important to education and academic performance. The perception that parents have similar ability as their children influences their children’s self-concepts, motivation, and, therefore, their performance (Simpkins et al., 2015). However, Jeynes (2005, 2012) pointed out the necessity of deepening the analysis of the role of parent involvement in the education of underachieving students.

McCoach and Siegle (2003a) attribute some of the differences between underachieving and non-underachieving students in students’ attitudes toward school and teachers. Gifted achieving students show differences in attitudes toward school, attitudes toward teachers, motivation/self-regulation, and goal valuation in comparison with gifted underachieving students. The findings obtained by Miñano et al. (2014) found that underachieving students showed the lowest levels of academic self-perception, attitudes toward school, attitudes toward teachers, motivation/self-regulation, and goal valuation.

Social factors, such as peer acceptance, may also promote achievement and underachievement (Reis and McCoach, 2000); negative peer attitudes can often explain underachievement. Negative attitudes of peers are usually related to underachievement, while popularity is often related to greater motivation, greater feelings of belonging at school, and higher academic performance (Wentzel et al., 2005).

### The Present Study

The first objective of this study was to compare these differences between underachieving and non-underachieving students using the standardized difference, the residual of regression, and the Rasch method of identification of underachieving students. With respect to giftedness, the identification methods of gifted students have generated a great deal of discussion (Brown et al., 2005). In the process of identification, a number of methodological aspects have been included, such as description of indicators, ways of obtaining information, and measurement questions (Heller and Schofield, 2008).

In relation with this objective, it is hypothesized (H1) that significant differences exist in the percentage of underachieving gifted students between the Rasch method and the other two methods (the simple difference method and the regression method).

The second objective was to examine the differences of educational capital and learning capital resources between underachieving and non-underachieving gifted students, which include personal, family and social variables. According to the literature, there are diverse reasons for underachievement as a school or family adjustment-related problem (Baker et al., 1998; McCoach and Siegle, 2003b) or personal attribute, such as low motivation or low self-concept (Reis and McCoach, 2000; Peixoto and Almeida, 2010; Dunlosky and Rawson, 2012; White et al., 2018). Baker et al. (1998) proposed a three-factor model to explain underachievement in American adolescent students and found that the variables that made the greatest contribution to the explanation of the differences between high- and low-performance students were self-regulation strategies, ability self-perception, and teacher-student relations (quality). Knowledge of these different characteristics is important to reverse underachievement (Renzulli and Reis, 1997; Chan, 1999, 2005).

With respect to the existing differences between underachieving and non-underachieving gifted students, it is expected that underachieving gifted students have significantly minor scores than non-underachieving gifted students on all of the studied variables (H2a), with the exception of achievement goals, social reinforcement goals, and general social self-concept, on which they are expected to have significantly higher scores (H2b).

## Materials and Methods

### Participants

This study used random cluster sampling with schools as the sampling unit, focusing on southeastern Spain. A total of 1,400 students in the first and second years of compulsory secondary education participated. Of those, 81.4% were enrolled in public school and 18.6% were enrolled in private school. Childhood socioeconomic status (SES) was established based on parents’ occupations, family incomes, and educational histories. There was a wide range of SESs; middle-class children made up the majority.

With reference to gender, 51.2% were boys and 48.8% were girls; the gender makeup in the national student population was 51.3% boys and 48.7% girls, and a chi-square test showed no gender differences between the sample and the population (χ2 = 0.29, df = 1, *p* > 0.01).

From the total sample, 164 participants with an IQs of 120 or higher (as measured by a test of intelligence) were selected, taking as reference the national normative published in the test manual. This subsample accounted for 11.71% of the total sample. Of these 164 students, 95 (57.9%) were males and 69 (42.1%) were females. There were statistically significant differences between the percentage of males and the percentage of females (χ2 = 4.12; *p* = 0.04).

### Measures

#### General Intellectual Ability

General intellectual ability was estimated using the Battery of Differential and General Abilities (BADyG) (Yuste et al., 2005), which evaluates students’ capacities and academic abilities using 192 items. Each item has five response options (only one correct response option) and offers a general intelligence quotient (IQ). The Cronbach’s alpha of the total IQ was 0.83.

#### Self-Concept

Marsh’s (1990) Self-Description Questionnaire (SDQ-II), which was adapted into Spanish (the Self-Concept Evaluation Scale for Adolescents [ESEA-2]) by González-Pienda et al. (2002), was employed to evaluate self-concept. This instrument comprises 70 items grouped into 11 self-concept dimensions, which are then grouped into three general dimensions; these were used in the present study and include general academic self-concept, general social self-concept, and general private self-concept. In the authors’ validation, all Cronbach’s alpha values were between 0.73 and 0.91. The answers were given on a 6-point Likert scale (1 = totally disagree, 6 = totally agree) to indicate the degree of agreement or disagreement with each statement.

#### Goal Orientation

García et al. (1998) Academic Goal Questionnaire (CMA), which is a Spanish adaptation of the Achievement Goal Tendencies Questionnaire by Hayamizu and Weiner (1991), was used to evaluate goal orientation. This instrument comprises 20 items grouped into three goals: learning, performance, and reinforcement. The answers are given on a 5-point Likert scale (1 = never, 5 = always), depending on the frequency with which the subject feels the statement to be true. In our sample, the Cronbach’s alpha values were 0.75, 0.72, and 0.85 for each of the three goals, respectively.

#### Learning Strategies

Learning strategies were measured using the Learning Strategies Questionnaire (CEA), produced by Beltrán et al. (2006), which evaluates four large scales. We only used the elaboration of information, personalization, and meta-cognition scales. To evaluate these three scales, students answered 50 items on a 5-point Likert scale (1 = completely false, 5 = totally true), indicating the degree to which each strategy was applicable to their own learning. We obtained Cronbach′s alpha values of 0.71–0.87.

#### Attitudes to School and Teachers

The Spanish adaptation of the School Attitude Assessment Survey-Revised (SAAS-R) by Miñano et al. (2014) was utilized to measure attitudes to school and teachers. The instrument was originally designed by McCoach and Siegle (2003b). The scale is made up of 35 items answered on a 7-point Likert scale; it measures five factors: AS, Academic Self-Perception, which explored students’ perception of the academic ability; ATT, Attitudes toward Teachers, which consisted of the students’ self-reported interest in their teachers and classes; ATS, Attitudes toward School, which consisted of the students’ self-reported interest in and affect toward school; GV, Goal Valuation, employed to measure students’ valuing of the goals of school; and M/S, Motivation/Self-Regulation, including the strategies employed to show high level of interest and to regulate cognition and effort (Pintrich and De Groot, 1990, p. 33). The reliability, or Cronbach’s alpha, obtained in the sample of 1,400 Spanish secondary school students was 0.86, 0.87, 0.90, 0.85, and 0.90 for each of the five factors, respectively.

#### Popularity

The popularity variable was measured using the BULL-S, as elaborated by Cerezo (2000). This instrument comprises 15 items. In this study, we used only the first four (“who would you choose as a classmate?,” “who would you not choose as a classmate?,” “who do you think has chosen you?,” “who do you think has not chosen you?”) to extract an index of peer acceptance (popularity).

#### Parent Involvement

The Parental Involvement Questionnaire (CIF) was used to evaluate the participation of parents. This questionnaire was created by our research group. Through this questionnaire, the students reported their perceptions of parental participation and monitoring and the importance that their parents place on the educational process. The instrument comprises 20 items grouped into four factors: (a) perception of support, planning, and interest in scholastic development (“I believe that my parents help me with my studies as much as they can”); (b) parental expectations (“my parents believe I can continue on to pursue post-compulsory education, i.e., high school or intermediate vocational training”); (c) school relations (“my parents regularly attend parent-tutor meetings”); and aid with homework (“my parents assist me with questions, homework, internet research, etc.”). Students answered the items on a 5-point Likert scale (1 = never or hardly ever, 5 = always or mostly), indicating the frequency that each statement is true. Cronbach’s alphas were 0.70, 0.65, 0.65, and 0.71 for each of the four factors, respectively.

#### Academic Achievement

To measure academic achievement, the mean GPAs from seven mandatory courses were employed. The courses registered were Spanish Language and Literature, Natural Sciences, Catalan Language, Social Sciences, Mathematics, English, and Technology. Grades from Art Education and Physical Education were discarded because of their lack of unidimensionality and also to investigate differences according to gender in this sample (Veas et al., 2017). The student scores showed high reliability, with a Cronbach’s alpha value of 0.94.

### Procedure

Mandatory consent was first obtained from the administrative staff and school boards of the schools, and the parents or legal guardians of the students then provided written informed consent. Data collection took place at the schools throughout the second trimester of the school year and during normal school hours over 4-h sessions. This study was approved by our Institutional Review Board and followed the ethical standard of the 1964 Helsinki Declaration and its later amendments or comparable ethical standards.

### Data Analysis

The simple standardized difference method was calculated based on the discrepancy between the standardized performance score and the standardized ability score. The students with a difference in punctuation lower than −1 were identified as underachieving. Secondly, the regression method was performed, with total IQ from the BADyG as the predictor and average grade of each student as the criteria. Students showing residual punctuation lower than −1 were identified as underachieving. SPSS version 21.0 software was used for both methods.

For the identification of underachieving students with the Rasch method, IQ scores from the BADyG and school grades were analyzed employing Winsteps version 3.81 statistical software (Linacre, 2011), and the estimates were based on the joint maximum likelihood (Linacre, 2012). Once fit indices from both measures have been obtained, the Rasch model allows for testing the hypothesis that two tests measure the same underlying construct (Bond and Fox, 2007). The procedures and results of these analyses are described in detail by Veas et al. (2016a,b).

To compare the profiles of the underachieving and non-underachieving students, a GLM (General Linear Model) was performed, which is a widely used procedure in profile analysis (Tabachnick and Fidell, 2007). Because not all of the variables were measured on the same scale, all of the scores were converted into z scores. Once the sample sizes were unequal, homogeneity (Box M) was tested. These analyses were performed with SPSS version 22.0.

## Results

Table 1 shows the number and percentage of underachieving and non-underachieving students identified by each method within the sample of high-ability students with IQs equal or superior to 120. From the 1,400 secondary school students who composed the sample of participants, 164 (11.71%) had IQs of 120 or above. Of these 164 high-ability students, 95 (57.9%) were male and 69 (42.1%) female, which was a slightly significant difference in percentage (χ2 = 4.12, *p* = 0.04).

**TABLE 1:** Descriptive statistics of the high-ability underachieving and non-underachieving students identified with the three statistical methods.

	**Method**								
	
	**Difference**			**Regression**			**Rasch**		
			
	**Frequency**	**Mean**		**Frequency**	**Mean**		**Frequency**	**Mean**	
						
		**IQ**	**Ach.**		**IQ**	**Ach.**		**IQ**	**Ach.**
Underachieving	42(25.6%)	129	6.87	18(11.0%)	126	5.76	24(14.6%)	125	6.10
Non-underachieving	122(74.4%)	125	8.84	146(89.0%)	126	8.48	140(85.4%)	126	8.54
	164(100%)			164(100%)			164(100%)		

*Ach., academic achievement; IQ, intellectual quotient.*

As can be observed, the numbers and percentages of subjects identified as underachieving were considerably different depending upon method of identification, becoming statistically significant (Cochran *Q* = 34.66, *p* = 0.001). The standardized difference method identified a greater number of underachieving students than the regression and the Rasch methods; the Rasch method identified a smaller number of underachievers in this high range of ability.

Three profiling analyses were performed to differentiate between underachieving and non-underachieving students; one was conducted for each of the identification methods in the personal, family, and social variables.

A univariate analysis of variance (ANOVA) of repeated measures and a multivariate analysis of variance (MANOVA) were performed in each analysis.

In the results of the ANOVA performed on the scores of subjects identified with the standardized difference method, Mauchly’s test did not confirm sphericity for the DV matrix (*W* = 0.001; χ2 = 1023.57, df = 170, *p* = 0.001); therefore, the degrees of freedom for the within-subjects test were corrected using Epsilon correction values. Once these corrections had been made, the *F* ratio for the flatness test was significant (*F* = 9.45, *p* = 0.001), indicating that there were differences between the variables within each group. In the test for parallelism – interaction, variables by group indicated that the profiles were different across groups (*F* = 37.80, *p* = 0.001).

To analyze whether significant differences existed between the variable scores of underachieving and non-underachieving students, the level test was conducted; this showed that the variable means for each group were significantly different one another (*F* = 40.82, *p* = 0.001).

Since the univariate analysis did not fulfill the sphericity assumption, the results of the multivariate analysis were included. For within-subjects effects, the Wilks Lambda was significant (λ = 0.42, *F* = 11.03, *p* = 0.001). The interaction variables by group were also significant (λ = 0.70, *F* = 3.35, *p* = 0.001).

**FIGURE 1:**
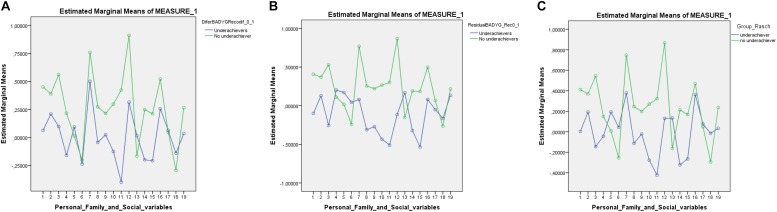
Profiles of high-ability underachieving and non-underachieving students identified by the standardized difference **(A)**, residual **(B)**, and Rasch **(C)** methods in personal, family, and social variables.

Figure 1 shows the profiles of the gifted underachieving students group and the gifted normally achieving students group.

A *t*-test for independent groups was performed to evaluate whether specific variables showed statistically significant differences between groups (the underachieving and non-underachieving students groups). Table 2 shows the descriptive statistics (means and standard deviations), *t* statistics, and significance of differences (*p*) for each of the identification methods employed. The results showed significant differences in most of the personal, family, school, and social variables. Underachieving students obtained lower scores in elaboration and metacognition strategies, learning goals, academic self-efficacy, attitudes to teachers, goal values, self-regulation, general academic self-concept, general private/personal self-concept, and perception of parents’ support compared to the non-underachieving students.

**TABLE 2:** Descriptive statistics and mean differences between underachieving and non-underachieving high-ability students identified by the three methods.

**Method**	**Simple standardized difference**				**Residuals of regression**				**Rasch model**				
			
	**Under $\bar{x}$**	**Non-under $\bar{x}$**	***t***	***p***	**Under $\bar{x}$**	**Non-under $\bar{x}$**	***t***	***p***	**Under $\bar{x}$**	**Non-under $\bar{x}$**	***t***	***p***	**No. of**
	***(SD)***	***(SD)***			***(SD)***	***(SD)***			***(SD)***	***(SD)***			**diff.**
**Variable**													
1	0.06 (1.01)	0.45 (0.89)	–2.34	0.02	−0.09(0.90)	0.40 (0.92)	–2.19	0.03	0.01 (0.84)	0.41 (0.94)	–1.99	0.04	3
2	0.20 (1.14)	0.39 (0.93)	–1.01	0.31	0.12 (1.06)	0.37 (0.98)	–0.97	0.33	0.19 (0.93)	0.36 (1.01)	–0.80	0.42	–
3	0.09 (1.16)	0.56 (0.97)	–2.52	0.01	−0.25(1.18)	0.52 (0.99)	–3.10	0.01	−0.14(1.07)	0.54 (1.01)	–3.07	0.01	3
4	−0.15(0.99)	0.21 (1.05)	–2.01	0.04	0.20 (0.74)	0.11 (1.08)	0.46^a^	0.64	−0.04(0.83)	0.14 (1.08)	–0.82	0.41	1
5	0.09 (0.94)	0.01 (1.11)	0.41	0.67	0.17 (0.96)	0.01 (1.08)	0.57	0.56	0.19 (0.99)	0.01 (1.08)	0.78	0.43	
6	−0.23(1.31)	−0.20(1.29)	–0.14	0.88	0.04 (1.02)	−0.24(1.30)	0.88	0.38	0.04 (1.07)	−0.25(1.33)	1.03	0.30	–
7	0.50 (0.83)	0.76 (0.73)	–1.96	0.05	0.08 (0.91)	0.76 (0.71)	–3.72	0.01	0.37 (0.79)	0.74 (0.75)	–2.20	0.02	3
8	−0.04(0.95)	0.27 (0.82)	–2.06	0.04	−0.31(1.02)	0.25 (0.83)	–2.65	0.01	−0.11(0.98)	0.24 (0.84)	–1.88	0.06	2
9	0.02 (1.04)	0.21 (0.80)	−1.07^a^	0.28	−0.27(1.08)	0.22 (0.82)	−1.86^a^	0.07	−0.02(1.02)	0.19 (0.84)	–1.14	0.25	–
10	−0.12(1.18)	0.29 (0.59)	−2.22^a^	0.03	−0.43(1.40)	0.26 (0.66)	−2.08^a^	0.04	−0.27(1.26)	0.26 (0.66)	−2.07^a^	0.04	3
11	−0.39(1.07)	0.42 (0.79)	−4.54^a^	0.01	−0.50(0.84)	0.30 (0.92)	–3.56	0.01	−0.42(0.94)	0.32 (0.90)	–3.70	0.01	3
12	0.31 (0.94)	0.91 (0.59)	−3.85^a^	0.01	−0.11(0.79)	0.86 (0.66)	–5.80	0.01	0.13 (0.82)	0.86 (0.67)	−4.13^a^	0.01	3
13	0.01 (1.26)	−0.16(0.95)	0.98	0.32	0.16 (1.37)	−0.15(0.99)	1.23	0.21	0.13 (1.23)	−0.16(1.01)	1.28	0.20	–
14	−0.19(0.95)	0.24 (0.86)	–2.80	0.01	−0.32(0.91)	0.19 (0.89)	–2.28	0.02	−0.32(1.04)	0.21 (0.86)	–2.71	0.01	3
15	−0.20(1.12)	0.21 (0.89)	–2.44	0.01	−0.53(1.20)	0.18 (0.91)	–3.02	0.01	−0.26(1.16)	0.16 (0.92)	–2.03	0.04	3
16	0.25 (0.92)	0.52 (0.51)	–1.76	0.08	0.08 (1.09)	0.49 (0.56)	−1.58^a^	0.13	0.36 (1.02)	0.46 (0.56)	−0.49^a^	0.62	–
17	0.06 (0.84)	0.04 (0.90)	0.09	0.92	−0.05(0.83)	0.06 (0.89)	–0.52	0.59	0.07 (0.79)	0.05 (0.90)	0.13	0.89	
18	−0.13(0.85)	−0.29(0.82)	1.01	0.31	−0.16(0.86)	−0.26(0.82)	0.47	0.63	−0.01(0.93)	−0.29(0.80)	1.52	0.13	
19	0.03 (1.04)	0.26 (1.13)	–1.16	0.24	0.13 (1.10)	0.21 (1.11)	–0.29	0.76	0.03 (1.12)	0.23 (1.11)	–0.82	0.41	–

*Variables: 1, elaboration strategies; 2, personalization strategies; 3, metacognition strategies; 4, learning goals; 5, social reinforcement goals; 6, achievement goals; 7, academic self-efficacy; 8, attitude toward teacher; 9, attitude toward school; 10, goal values; 11, self-regulation; 12, general academic self-concept; 13, general social self-concept; 14, general private self-concept; 15, parent support; 16, expectations; 17, school relations; 18, time on homework; 19, popularity. ^*a*^Equal variances not assumed.*

In the analysis of data obtained with the residual scores of the regression technique, again, Mauchly’s sphericity test did not confirm sphericity for the DV matrix (*W* = 0.002; χ2 = 1006.79, df = 170, *p* = 0.001); therefore, the degrees of freedom for the within-subjects test were corrected using Epsilon correction values. After that, the *F* ratio for the flatness test was significant (*F* = 2.97, *p* = 0.001), indicating that there were differences between the variables within each group. More importantly, the test for parallelism – interaction variables by group indicated that the profiles were different across groups (*F* = 3.64, *p* = 0.001). The profiles of both groups are shown in Figure 1B.

The level test showed that the means of the motivational and attitudinal measures were significantly different in each group (*F* = 10.07, *p* = 0.002).

The results of the MANOVA indicated that regarding within-subjects effects, the Wilks Lambda was significant (λ = 0.66, *F* = 4.06, *p* = 0.001). The interaction variables by group were also significant (λ = 0.72, *F* = 3.04, *p* = 0.001).

To assess which variables presented statistically significant differences between the groups, a *t*-test for independent groups was performed. The results of this analysis are shown in Table 2. The results showed differences in the same variables as in the standardized difference method, with the exception of learning goals, which showed no significant differences between groups.

The ANOVA performed on the data obtained from the subject identified by the Rasch method showed that Mauchly’s sphericity test did not support sphericity (*W* = 0.002, χ2 = 1011.82, df = 170, *p* = 0.001); therefore, the degrees of freedom for the within-subjects test were corrected using Epsilon correction values. The *F* ratio for the flatness test was significant (*F* = 4.59, *p* = 0.001), indicating that there were differences between the different variables within each group. Further, on the test for parallelism – interaction, variables by group indicated that the profiles were different across groups (*F* = 3.32, *p* = 0.001). The profiles for both groups are shown in Figure 1C.

The level test also showed significant mean differences in the variables between groups (*F* = 6.07, *p* = 0.01).

The results of the MANOVA indicated that regarding within-subjects effects, the Wilks Lambda was significant (λ = 0.56, *F* = 6.33, *p* = 0.001). The interaction variables by group were also significant (λ = 0.72, *F* = 3.01, *p* = 0.001).

The *t*-test results presented in Table 2 indicate that significant differences were found for the same variables as in the residual regression method, with the exception of that related to attitudes toward teacher. Underachieving students had lower scores in elaboration and metacognition strategies, academic self-efficacy, goal values, self-regulation, general academic self-concept, general private/personal self-concept, and perception of parents support compared to non-underachieving students.

Although the Box’s M test did not show homogeneity of variance–covariance matrices in the MANOVA, the highest ratio of variance between groups did not exceed the 1:10 (Tabachnick and Fidell, 2007) in the analysis performed on the scores of subjects identified with the standardized difference method (1:6.45), residual regression method (1:6.17), or Rasch method (1:4.91).

Looking again at Table 2, it can be observed that in most cases, the differences obtained with either method occurred in the same variables. The exception was in learning goals, where the only differences between underachievers and non-underachievers occurred with the method of standardized differences and in the variable attitudes toward the teachers, in which differences between the students and the Rasch method do not occur.

## Discussion

The results allow us to respond to the research objectives, which were to examine the differences between underachieving and non-underachieving gifted students in individual, family, social, and school variables and compare these differences when different methods of identification of underachievement are used.

First, the percentage of participants identified as underachieving differed significantly, depending on the method of identification. In this sense, although a higher number of underachieving students were expected to be identified by the Rasch method (H1), both the standardized difference method and the regression method identified a similar number of students. This discrepancy may be due to a minor level of differences between gifted students in comparison with students from other ability ranges. However, given the lack of generalization of this method, further studies should be developed to explore psychometric properties according to students’ characteristics.

Although these results, which were obtained from among high-ability students, reveal the lack of consistency in the different operational definitions of underachievement, all three methods identified a significant percentage of underachieving students, similarly to other studies involving students with broader ability ranges (Phillipson, 2008; Veas et al., 2016a,b).

Second, the results showed statistically significant differences between underachieving and non-underachieving students in most of the variables studied, as is pointed out by recent revision studies on gifted underachievement (Siegle and McCoach, 2018; White et al., 2018).

Regarding learning strategies, these were used less by underachieving students, who reported minor use of elaborative and metacognitive strategies. These findings were comparable to those reported in studies on gifted underachieving students (Dowdall and Colangelo, 1982; McCoach and Siegle, 2003b; Colangelo et al., 2004), in which underachieving students showed decreases in these strategies. From this, we can conclude that learning strategies are a key variable to explain underachievement (Chiu et al., 2007; Yip, 2007).

With regard to motivation, the results showed that underachieving students reported lower scores in learning goals compared with non-underachieving students, whereas no differences in achievement goals or social reinforcement goals were shown. There are many studies showing lower levels of motivation in underachieving students (Schick and Phillipson, 2009; Dunlosky and Rawson, 2012; Preckel and Brunner, 2015).

High-ability underachieving students also showed worse academic self-perceptions, attitudes toward teachers, goal values, and motivation/self-regulation, as reported by McCoach and Siegle (2003a), who pointed out these variables in high-ability students with low achievement. Castejón et al. (2016) also found this in a sample with a broader range of ability.

With respect to self-concept, high-ability underachieving students showed lower general academic self-concepts and personal/private self-concepts. These results are similar to the studies by Preckel and Brunner (2015) and Rimm (2003). McCoach and Siegle (2003a, b), on the other hand, found lower general self-concepts but not lower academic self-concepts in gifted underachieving students. In the same way, McCoach and Siegle (2003a, b) found that underachieving students showed lower private/personal general self-concepts. In all of these works, underachieving students evidenced lower personal self-concepts.

In this case, the second hypotheses (H2a and H2b) are partially accepted, as underachieving gifted students showed higher scores on learning goals, but they did not score significantly higher on achievement goals and social reinforcement goals.

However, according to our hypotheses, underachieving students showed significantly higher scores than non-underachieving students in general social self-concept. Although the majority of the studies that analyze differences between gifted and non-gifted students in self-concept dimensions, gifted students showed significantly lower scores than the non-gifted ones in social self-concept (Zeidner and Shani-Zinovich, 2015). In this case, within a gifted sample, it has been shown that it is not a homogeneous group in this factor, and underachieving students showed considerably lower scores.

Differences in family factors also were found. High-ability underachieving students perceived lower parental support, although there were no significant differences in perceived parental expectations, relations of parents with the school, or reported time spent supporting homework compared to non-underachieving students. Parental expectations, parental support, and parent-school relationships seem to be good predictors of parental involvement and student achievement, as stated by some meta-analyses (Jeynes, 2005, 2012; Wilder, 2014). Also, higher time support is related to lower academic performance (Gonida and Cortina, 2014). Contrary to expectations, there were no significant differences in popularity between high-ability underachieving and non-underachieving students.

In sum, the profiles of the high-ability underachieving students showed minor use of elaboration and metacognitive strategies, less learning goal orientations, poor academic self-perceptions, minor attitudes toward teachers, minor self-regulation, lower academic and personal self-concepts, and lower perceptions of parent support in the educational process. In most cases, the differences obtained with any method used occurred in the same variables.

Knowing these characteristics is necessary for the design and implementation of programs aimed at reversing the low academic performance of high-ability underachieving students (Renzulli and Reis, 1997; Chan, 1999, 2005). Further, any educational intervention focused on reversing low academic achievement in high-ability underachieving students must focus simultaneously on these characteristics (Baum et al., 1995).

Taken together, these results showed high congruence between methods of establishing differences in the variables, despite their different operational definitions of underachievement. Regardless of the method employed, there were significant differences between underachieving and non-underachieving students in terms of individual and family characteristics; this was held true for the current study, which involved high-ability subjects, and in studies that included larger samples of participants with broader ranges of ability (Castejón et al., 2016). Therefore, the results obtained so far support the concept of underachievement and the characteristics of underachieving students, regardless of their capacity.

Considering the actiotope model of giftedness as a dynamic model, these results let us propose possible educational strategies to reverse underachievement. In the first place, although gifted students should have a clear intellectual potential, this capacity needs internal cognitive resources that resolve “how” to work with academic contents. In this area, learning strategies are crucial cognitive tools to be trained in from childhood. Thinking about how personal resources could be improved leads us to the second point, to create parenting-school communication bridges with similar patterns of interests and contexts. From the social educational capital perspective, many studies have concluded that student achievement is related more with intellectual stimulation in the home than to parental socioeconomic status (Woolley and Grogan-Kaylor, 2006).

Given these consistent results, it is clear that there is a need for constant interactions between family and teachers. Moreover, by knowing the parents’ perspectives on the factors that support the development of giftedness in their children, it is possible for gifted students to gradually internalize a positive motivation and self-concept (Heller, 2010). At the same time, it is important to consider the access of high-quality education for gifted students from an early age (Vialle, 2017). Apart from classical enrichment programs, and although unexplored in gifted students, possible useful interventions can be those under the funds of knowledge approach (González et al., 2005), focused on having teachers learn about family knowledge and skill that they can use to plan learning activities that connect the curriculum to family skill.

Finally, some limitations may be addressed. First, the present work involved a relatively low number of high-ability students as participants, which could prevent the appearance of significant differences in some variables. For this reason, future studies with larger numbers of high-ability students are needed with adequate sampling procedures to ensure representativeness. Second, longitudinal analysis is also be needed to explore the measures’ consistency at different time points. This would let us explore reciprocal relations among the variables.

## Data Availability Statement

The datasets generated for this study are available on request to the corresponding author.

## Ethics Statement

The research meets ethical guidelines, including adherence to the legal requirements of the study country. The present research was approved by the Ethical Committee of the University of Alicante.

## Author Contributions

RG-C: quantitative methods and theoretical review of the study. AV: theoretical review of the study. PM: theoretical review of the study and review of the references. J-LC: quantitative methods.

## Conflict of Interest

The authors declare that the research was conducted in the absence of any commercial or financial relationships that could be construed as a potential conflict of interest.
